# Significance of 1B and 2B domains in modulating elastic properties of lamin A

**DOI:** 10.1038/srep27879

**Published:** 2016-06-15

**Authors:** Manindra Bera, Sri Rama Koti Ainavarapu, Kaushik Sengupta

**Affiliations:** 1 Biophysics & Structural Genomics Division, Saha Institute of Nuclear Physics, 1/AF, Bidhannagar, Kolkata-700064, India; 2 Department of Chemical Sciences, Tata Institute of Fundamental Research, Mumbai, Homi Bhabha Road, Mumbai, 400005, India

## Abstract

Nuclear lamins are type V intermediate filament proteins which form an elastic meshwork underlying the inner nuclear membrane. Lamins directly contribute to maintain the nuclear shape and elasticity. More than 400 mutations have been reported in lamin A that are involved in diseases known as laminopathies. These mutations are scattered mainly in the lamin rod domain along with some in its C-terminal domain. The contribution of the rod domain towards the elasticity of lamin A molecule was hitherto unknown. Here, we have elucidated the significance of the 1B and 2B domains of the rod in modulating the elastic behavior of lamin A by single-molecule force spectroscopy. In addition, we have also studied the network forming capacity of these domains and their corresponding viscoelastic behavior. We have shown that the 1B domain has the ability to form a lamin-like network and resists larger deformation. However at the single-molecular level, both the domains have comparable mechanical properties. The self-assembly of the 1B domain contributes to the elasticity of the lamin A network.

The nucleus of the metazoan cell maintains its 3D morphology and rigidity with the help of filamentous elastic scaffold underlying the inner nuclear envelope^1^,^2^. This elastic scaffold is composed of lamin proteins which are classified as type V intermediate filament proteins (IFs)^3^. The nucleus is further tethered to the cytoskeleton. Nonequilibrium fluctuations in the cytoskeleton in response to external mechanical cues could be transmitted into the nuclear lamins via LINC complex^4^,^5^. It has been hypothesized that IFs act by offering resistance to mechanical strain due to their unique elastic properties and lamins also provide mechanical rigidity to the nucleus^6^,^7^. Nuclear lamins are two types: A and B^8^. A-type lamins comprising of lamin A and lamin C are transcribed from *LMNA* gene where lamin C is the splice variant of lamin A. B-type lamins, lamin B1 and lamin B2, are encoded by the *LMNB1* and *LMNB2* genes respectively^9^. It has been recently shown in a set of elegant studies that lamin A concentration varies greatly from hard tissue to soft tissue compared to the lamin B^10^ and that the nuclear stiffness is largely controlled by the lamin A^11^,^12^. Like all other IFs, nuclear lamins also have the same tripartite structure consisting of the short unstructured N-terminal head domain, a long α-helical central rod domain followed by another largely unstructured C-terminal domain with a single immunoglobulin-like (Ig) domain^13^. The α-helical central rod domain can further be divided into four regions designated as coils 1A, 1B, 2A and 2B, separated by linkers L1, L12 and L2 respectively. The central rod domain contains the characteristic heptad repeats which help lamins to form the coiled-coil dimers through intermolecular hydrophobic interactions^3^,^14^,^15^. From *in vitro* studies, it was shown that polarized arrays of the dimers interact in an antiparallel fashion to form apolar tetrameric protofilaments. The interaction of four lamin protofilaments leads to the formation of ~10 nm filaments^16^. The lamin A network is crucial in maintaining the nuclear shape, which is important in cell migration during developmental stage and in wound healing^17^. Moreover, several diseases such as dilated cardiomyopathy (DCM), Emery-Dreifuss Muscular Dystrophy (EDMD), Hutchinson-Gilford Progeria Syndrome (HGPS), Familial Partial Lipodystrophy (FPLD), Duchene Muscular Dystrophy (DMD) etc. result from the loss of the mechanical integrity of the nuclear lamina^18^,^19^. Over the past few decades, more than 400 mutations in the *LMNA* gene have been linked to these diseases, which are collectively known as laminopathies^20^,^21^,^22^. Laminopathies are linked by the common phenotypes of fragile and misshapen nuclei. According to the current knowledge, two hypotheses have been postulated to link lamin mutation and laminopathies in a cause and effect relationship. The structural hypothesis suggests that mutation in lamin A leads to a loss in the integrity of the nuclear lamina thus leading to its structural weakness. Hence, the nuclear integrity is perturbed in response to external mechanical forces as in the skeletal muscle for instance^23^. Gene regulation hypothesis assumes that mutations in the *LMNA* gene alter the regulation of gene expression during differentiation of tissues of mesodermal origin^24^. But these hypotheses are not mutually exclusive^25^. Here, we aim to elucidate the elasticity of the helical domain of lamin A in the light of the structural hypothesis. To dissect the origin of the elastic property of the lamin A network, we took two fragments of the rod domain of lamin A. It was earlier shown that the rod domain fragments are capable of forming short lamins or “mini-lamins”^26^. Interestingly, most of the laminopathic mutations occur in the rod domain, especially in 1B and 2B domains. Lamin 2B domain also possesses a stutter defect as suggested by the crystal structure^27^. As the full-length rod domain has the propensity to aggregate, we divided it into two distinct domains – 1B and 2B for this study. We established that each domain retained the capacity to form the network much like “half-lamin” or “mini-lamin”^26^. Subsequently, we performed single-molecule force spectroscopy pulling experiments to measure their elastic properties at the dimer level. Furthermore, we corroborated these results with the bulk viscoelastic measurements to show the elastic nature of these fragments. Although at the dimer level 1B and 2B showed similar elastic behavior, the assembled network of 1B domain proved to be more robust. In other words, the bulk 1B domain had higher elastic modulus reflecting higher load bearing capacity. To the best our knowledge, this is the first report where the individual contributions of 1B and 2B domains towards the elasticity of lamin A have been established.

## Results

### Lamin A heptad repeats and mini-lamin formation

**Figure 1:**
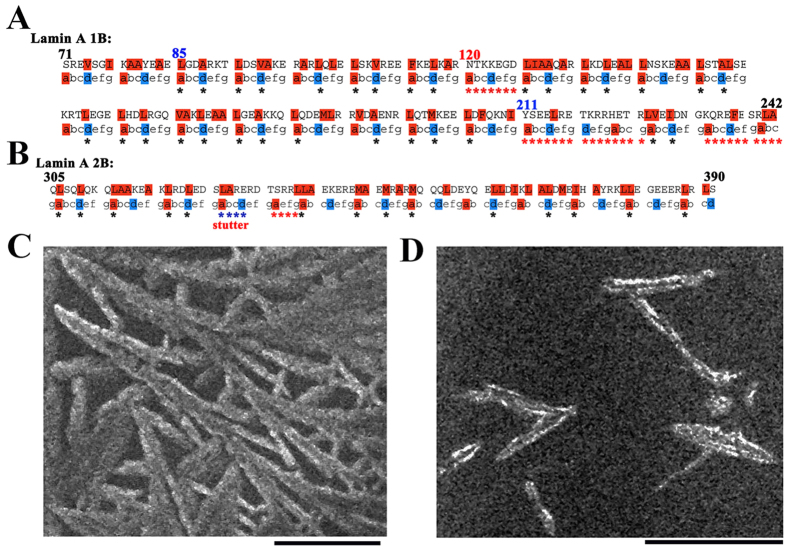
Heptad repeat pattern of lamin 1B and 2B and their network formation. Schematic representation of the heptad repeats for 1B and 2B are shown in (**A**,**B**) respectively. In 1B, there is a seven residue heptad repeat skip at position 120 and at the end from residue 211–242 heptad repeats are not feasible suggesting that it is a linker region, whereas for 2B at position 333 a four residue skip was found out suggesting a presence of a stutter. Blue stars for 2B represents the stutter defect as revealed by the crystal structure, red stars represent the mismatch in heptad I from our study. The heptad repeat calculations were performed by PCOILS/COIL server using MTIDK matrix with 14 residue window size. Seaview tool was used for representation. Scanning electron micrographs (SEM) are shown in (**C)** and (**D**) for 1B and 2B respectively. 1B domain was forming lamin-like network whereas 2B domain was forming only short lamins. The SEM images were taken in 4000X and 6000X magnification respectively. All the SEM experiments were performed using 10 mM Tris buffer, pH 8.5, 250 mM NaCl and 1mM DTT. 5 μM and 20 μM concentrations of protein were used for 1B and 2B respectively. Τhe scale bar is 5 μm.

Like all other IFs, lamin A proteins have the same basic tripartite structure^28^. Lamin A 1B domain is the largest domain and is considered as to be a hot spot for laminopathic mutations^29^. According to the lamin database (http://www.dmd.nl/lmna_home.html#protein)^30^, 1B domain originates from Tyr 81 and terminates at Thr 218. We analyzed the coiled-coil forming propensity of this region using PCOILS server^31^. Based on the heptad repeat formation prediction from COILS/PCOILS, we found out that 1B domain started from residue 85 and extended up to residue 210 (shown in Fig. 1A). But coiled-coil forming propensity was up to residue 227 which explained the coiled-coil nature of the linker region whereas N-terminal linker of the 1B domain showed less coiled-coil propensity compared to the C-terminal linker region. Surprisingly we observed that insertion of seven residues (NTKKEGD) at position 120 disrupted the regular coiled-coil heptad repeats. Similarly, we also examined the lamin A 2B domain in COILS/PCOILS server using the same MTIDK matrix. Crystal structure of the 2B domain already showed the stutter defect at position 327–330^27^ whereas we uncovered a similar defect at the 334–337 position because of the insertion of four residues (SRRL) (shown in Fig. 1B). However, lamin 1B domain contains 11 ideal heptad repeats where *a* and *d* positions of the heptad were occupied by a nonpolar residue (L, A or V). On the other hand, 2B domain contains only four heptad repeats. Lamin A forms a coiled-coil dimer through the intermolecular hydrophobic interactions of the coiled-coil regions and these dimers further can form the higher order structure by head-to-tail association. Different lamin fragments in both coil 1 and coil 2 regions can form the half-lamin or mini-lamin^26^. Initially, we checked whether the fragments could retain the ability to form the higher order oligomers. Hence, we generated these fragments, expressed and purified the proteins for experimental studies (Supplementary Fig. 1). Subsequently, scanning electron micrographs (SEM) of lamin 1B and 2B domain proteins demonstrated that both domains independently could assemble into higher order structures (shown in Fig. 1) as in the case of the half/mini-lamins described earlier^26^. 1B domain alone was sufficient to form a lamin-like meshwork and showed a propensity to bundle altogether whereas 2B domain formed small spindle-like fragments and did not form a crisscross network.

### Mechanical resilience of the half-lamin at dimer level

**Figure 2:**
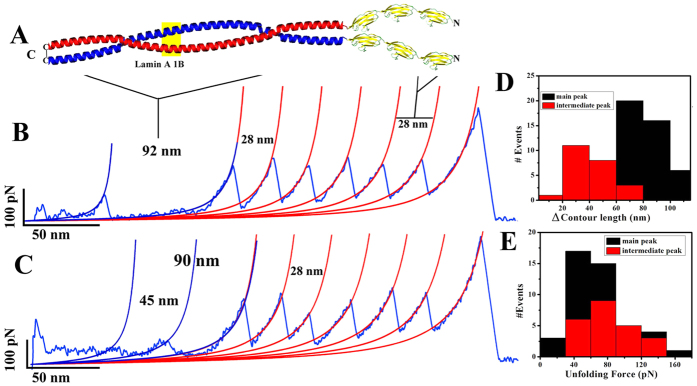
SMFS experiments of the coiled-coil 1B domain. (**A**) Cartoon represents the molecular construct used for the coiled-coil unzipping of 1B domain. The molecular construct for SMFS experiments was (I27)_3_-1B-1C. Representative force-extension (F-X) traces are shown in (**B**,**C**). (**B**) is representing the end-to-end two-state unzipping of the 1B dimer whereas (**C**) is showing the three-state unfolding. In both cases, the lamin force peak followed by six I27 force peaks with ~28 nm spacing in the F-X traces. Histograms for the contour length increment and the unfolding forces are shown in (**D**) and E respectively. Average uncoiling force for the 1B dimer is ~70 pN and contour length increase (ΔL_c_) is ~85 nm. Almost 60% dimers were unfolding via several intermediates. Average unfolding force and (ΔL_c_) for the intermediate peaks were ~84 pN and 45 nm respectively. WLC fits are also shown in FX curves. All the SMFS experiments for 1B were done at ~5 μM concentration in 25 mM Tris-Cl (pH 8.5), 250 mM NaCl buffer at 25 °C.

We have thus shown that both 1B and 2B domains can assemble into higher order structures. Now it would be interesting to investigate their mechanical resilience at their single-molecule level. We performed pulling experiments on lamin 1B and 2B by single-molecule force spectroscopy (SMFS) to understand the mechanical properties of their dimers. To demonstrate distinct coiled-coil unzipping, we engineered tandem titin I27 domains^32^,^33^,^34^(shown in Fig. 2A). We engineered three I27 domains followed by the lamin rod domains. We introduced a cysteine residue at the C-terminal of the lamin rod domains. This dimer formation will thus be stabilized by the C-terminal cys-cys disulfide bond. Hence, the resultant molecules were (I27)_3_-1B-(Cys) and (I27)_3_-2B-(Cys) which would be henceforth referred to as (I27)_3_-1B-1C and (I27)_3_-2B-IC. Upon unfolding of these disulfide-linked dimers, we would expect the resultant unfolding pattern of corresponding six I27 domains and a coiled-coil unzipping of the lamin domain. We ensured the dimer formation by running the proteins in a non-reducing SDS-PAGE gel (see supplementary Fig. 1A). When pulled, both the 1B and 2B constructs gave saw-tooth patterns with six force peaks with contour length spacing of ~28 nm and peak forces ~200 pN, which corresponded to I27 repeats, and a lamin unzipping peak at the beginning of the force-versus-extension (F-X) traces. As both the molecules have the self-assembling property, we also obtained large forces for the lamin. For analysis, we considered only F-X traces which contained saw tooth patterns with at least four I27 force peaks as this guarantees the mechanical stretching of the lamin dimer. I27 domains were unfolded at ~200 pN force with regular ~28 nm spacing which is in excellent agreement with previous data^34^,^35^. We performed all the SMFS experiments with 1000 nm/sec pulling speed and all the force-extension curves were fitted to the worm-like chain (WLC) model^36^,^37^. In SMFS experiments, both the 1B and 2B dimers showed a large variation in the increase in contour length (ΔL_c_) reflecting the flexibility and heterogeneity in the local structure at the dimer level^38^. Lamin A 1B dimer exhibited unfolding at ~70 pN force with ~82 nm increase in contour length. However, almost 60% 1B domains unfolded via several intermediate pathways, which is in agreement with the PCOILS/COILS heptad repeat calculations where we found that strong inter- helical interactions occur from the residue 85–210.We expected a theoretical ~90 nm increase in contour length (ΔL_c_) based on the dimer formed from 126 amino acids in the lamin fragment used in the SMFS experiments^39^. Almost 40% of the intermediates yielded a peak around 26 nm which might be due to an irregularity in the heptad repeats at position 120. A significant percentage of the intermediates stretched at ~42 nm which might be due to the unzipping of each α-helical region. Furthermore, we also obtained a significant variation in the contour length increase for the main peak. We showed that C-terminal linker region of the 1B domain also had the ability to form the coiled-coil helix. Hence, we observed the variation; however inter-helix stability of this linker region was not strong enough to produce a strong peak. The average unfolding force of the intermediates was ~84 pN, which points to a probable strong intermediate, compared to the main unfolding force peak.

**Figure 3:**
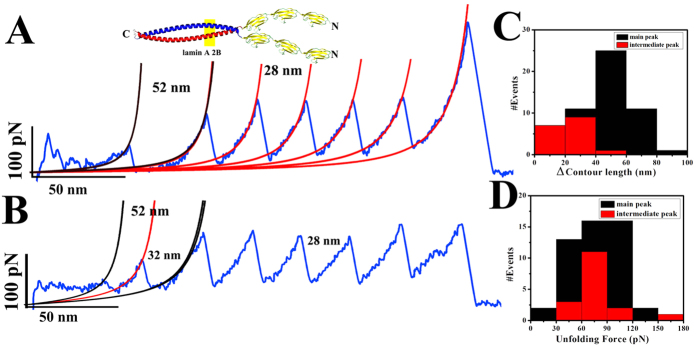
Coiled-coil unzipping of 2B domain. Panels (**A,B**) show representative FX traces for the two-state and three-state unfolding of 2B. Similar to 1B domain lamin 2B dimers were also unfolding via several intermediates. The molecular design for SMFS experiment was (I27)_3_-2B-1C. Average unfolding forces for both the intermediate and main peaks were similar, at 75 pN. Cartoon picture represents the dimer of the 2B domain. In (**A**) first unfolding peak with 52 nm contour length increase corresponds to lamin 2B domain two-state unfolding and I27 domains are folding with 28 nm spacing at ~180 pN force. Panel (**B**) is representing the three state unzipping events. In (**C**,**D**) histograms for the contour length and unfolding force analysis has been shown respectively. The black bar is representing the value for the main peak whereas red is depicting the intermediate peaks. Average contour length increments for the main and intermediate peaks were ~52 nm and 25 nm, respectively. Almost 40% molecules were unzipping via an intermediate pathway. All these unfolding experiments for the 2B domains were done at 5–10 μM concentration using 25 mM Tris-Cl (pH 8.5), 250 mM NaCl buffer at 25 °C.

We observed similar results for 2B domain also (shown in Fig. 3). Lamin 2B dimer unfolded at ~75 pN forces and increase in contour length was around 51 nm. 2B domain forms the coiled-coil dimer from residue 313–386 as suggest by the crystal structure. Hence, theoretically unzipping of the dimer should give rise to 52 nm increase in end-to-end. Interestingly, dimer of the lamin 2B domain also unfolded via an intermediate pathway. Lamin 2B domain possesses a stutter defect at 327–330 position; hence, an intermediate peak at ~15 nm was due to the unfolding of the 313–327 residues. Almost 50% of the intermediates unfolded at ~25 nm which might be the result of uncoiling of each α-helical strand and the average unfolding forces of the intermediates were also at ~75 pN. Persistence length and contour length data of these force-extension curves is given in Table 1. We also compared the elastic properties of the mutant 1B dimers (E161K and K97E) by SMFS (shown in the supplementary Fig. 2). 1B E161K dimer also unfolded via an intermediate pathway and at similar force range compared to the wild type. As shown by our group previously, K97E was prone to aggregation^40^. This was true for the K97E-I27 chimeric protein as well and we could not get many single-molecule pulling recordings on dimers of this protein. Nevertheless, from the limited data on the protein, we conclude that the K97E dimer unravels at very low forces without any discernible force peaks above the noise level.

### Thermal unfolding of the half-lamin

In order to corroborate the mechanical unzipping of the 1B and 2B at dimer level, we studied thermal unfolding in the bulk. We performed differential scanning calorimetry (DSC) from 10 °C to 85 °C at a scanning rate of 30 °C/hr. Both the unfolding events were endothermic. We calculated the calorimetric enthalpy (∆H_cal_) by integrating the area under a DSC thermogram which is described by the following equation.




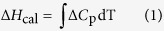




where, ∆H_cal_ and ∆C_p_ represent calorimetric enthalpy and change in molar heat capacity respectively.

**Figure 4:**
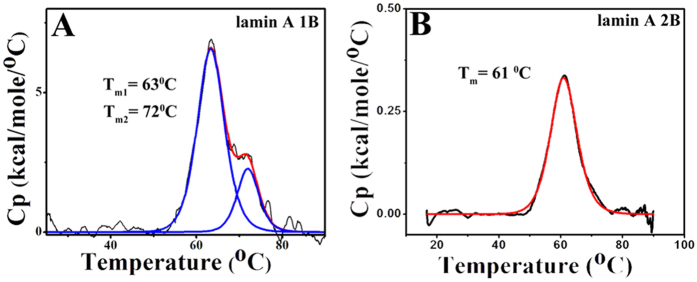
Thermal denaturation of lamin 1B and 2B domain. The thermal denaturation studies of the 1B and 2B domains were performed using differential scanning calorimetry in panels (**A** & **B**). 1B domain (panel **A**) was unfolding via an intermediate pathway whereas 2B (panel **B**) was denatured via the two-state mechanism. The first transition temperature for the 1B was at 63 °C and the final transition was at 72 °C, but 2B domain unfolded at 61 °C. Black curves represent the thermogram whereas red and blue curves were depicting the two-state and three-state fitting. All the fittings were performed using origin 6.0. Thermal unfolding experiments were performed at 20 μM and 100 μM concentration for 1B and 2B respectively at a scan rate of 30 °C/hr in 25 mM Tris-Cl (pH 8.5), 250 mM NaCl buffer.

Lamin 1B domain unfolding was fitted with a three-state model resulting from well separated dual transition temperatures of 63 °C and 72 °C whereas 2B domain unfolded at 61 °C via two-state process (shown in Fig. 4). Calorimetric enthalpy for the 2B unfolding process was ~3.7 kcal/mole but for the 1B domain, ∆H_cal_ was very large compared to the 2B domain. For the 1B domain ∆H_cal1_ and ∆H_cal2_ were ~56 and ~15 kcal/mol respectively which suggested that helix-coil transition required higher energy for the 1B domain. This points to the fact that 1B formed a higher level of assembly compared to the 2B. Hence, 1B required more energy for dissociation. The proteins precipitated after the transition temperature and hence reversibility could not be checked. The transition temperatures and the enthalpy values are shown in Table 2.

### Viscoelastic behavior of the half-lamins

It is now challenging to unravel the effect of the distinct assembly mechanisms of 1B and 2B on their corresponding viscoelastic behavior. In retrospect, detailed studies from our group had already established the viscoelastic nature of the full-length lamin A protein in the context of some of its mutations implicated in DCM^41^. Following on the same lines, we investigated the viscoelastic property of 1B and 2B domains to understand the origin of the elasticity of lamin A molecule due to individual contribution of different moduli. To understand the origin of the viscoelasticity we performed the rheological experiments applying the sinusoidal strain on these lamin molecules of different concentrations. A pure elastic material shows no frequency dependence in elastic response, however, this not true for the viscoelastic material. To study the viscoelastic properties, most commonly a sinusoidal strain was applied. Theoretically, the applied strain can be represented as **ɤ (t)** = **ɤ**_**o**_ **sin (ωt)**, so that the resultant stress becomes **σ (t)** **=** **σ**_**o**_**sin (ωt** **+** **δ)**. This resultant stress can be analyzed by decomposing the two waves of the same frequency (ω); one is in phase and another 90° out of phase. The stress can be written in the form of the complex dynamic modulus.




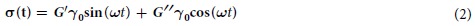




where, σ (t), G′ and G″ represent the applied stress, shear and loss moduli respectively




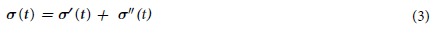







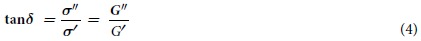




**Figure 5:**
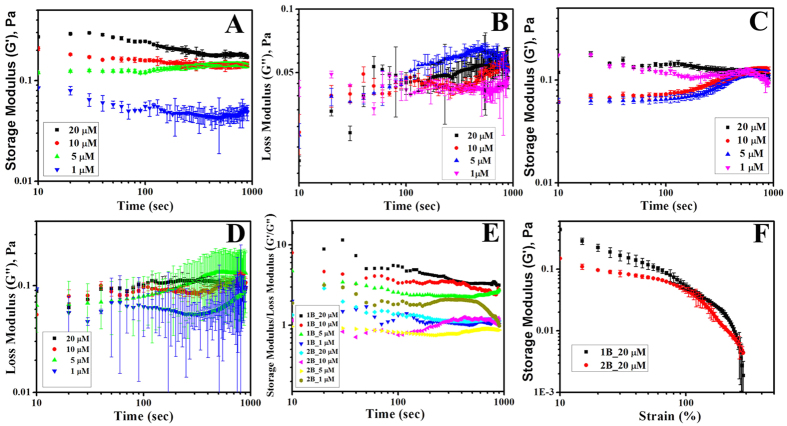
Viscoelastic measurements of mini-lamins. The elasticity of the half-lamins formed by 1B and 2B domains were shown using rheology. The elastic storage moduli of 1B and 2B domain at 1, 5, 10 and 20 μΜ concentrations are shown in panel A and C whereas corresponding elastic loss moduli are depicted in panel B and D respectively. Ratios of the corresponding storage moduli to loss moduli are shown in panel E. Network yield-up experiments varying strain from 0–500% are shown in panel F.

The ratio of the loss modulus to the storage modulus gives an idea about the viscoelastic nature of the fluid. We performed the oscillatory shear experiments with the preassembled half-lamin molecules. We applied constant oscillatory shear of strain amplitude 1% at an angular frequency of 5 rad/s for 1000 sec on the network formed by the half-lamins at various concentrations. Interestingly, we noticed that after 100 sec, both the storage and loss modulus of the networks reached the saturation level (data shown in Fig. 5). Storage modulus or G′ varied linearly with concentration for 1B network compared to the loss modulus which suggested that the network formed by the 1B possessed higher potential energy. But for the 2B domain G′ value was more or less similar for both 1 and 20 μM concentration which suggested that in both concentrations lamin 2B could not form the network. This was in excellent agreement with our scanning electron microscopy data, where it was shown that the only 1B has the capacity to form lamin-like network but 2B can form only short filaments. The ratio of the G′ to G″ suggested that 1B domain indeed formed the network as it was behaving as viscoelastic solid. We focused on concentrations between 1 and 20 μM based on circular dichroism data where we observed that inflection point for the 2B domain was at ~1 μM. Thus assembly for 2B domain might be initiated at 1 μM concentration and at 20 μΜ it was already well saturated (data shown in supplementary Fig. 3). The values for the G′ and G″ of both the networks at 1 μΜ concentration were comparable suggesting the sol-gel transition point. 1B network showed three times more solid-like nature compared to the network formed by the 2B domain at a concentration of 20 μM. However, we also examined the load bearing capacity of both the networks. We applied the force on the network with varying strain from 0–500%. We observed that 1B domain had much more load bearing capacity. The network formed by the 1B melted around 200% of strain whereas 2B melted early, at 100% of the strain (shown in Fig. 5F). Therefore, 1B domain was more important in forming the resistant viscoelastic meshwork formed by the lamin A molecule.

## Discussion

**Figure 6:**
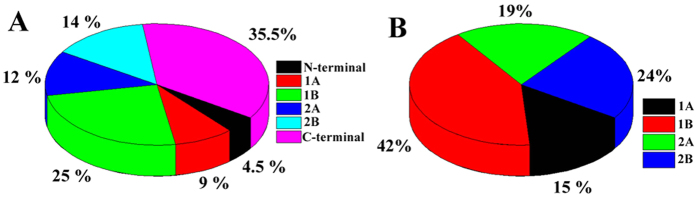
Statistical analysis of the lamin A mutation. A statistical analysis of the laminopathic mutation in the different domains of lamin A protein was performed based on the data available in the lamin mutation database (http://www.umd.be/LMNA/). Figure A represents the distribution of all the laminopathic mutations all over the lamin A molecule whereas Fig. B represents the same statistics confined to the rod domain only.

The filamentous elastic scaffold inside the cell consisting of nucleoskeleton and cytoskeleton has a major contribution in maintaining the cellular 3D morphology^42^. These networks play a major role in cellular mechanics by resisting the deformation in response to the external mechanical cue^43^. Elastic properties of the IFs enable them more to resist deformation due to mechanical strain^6^,^7^. Lamin A is such an intermediate filament protein of nuclear origin which is highly elastic in nature thereby maintaining nuclear and cellular homeostasis^44^. Lamin A inside the cell forms thick crisscrossed bundles and layers which provide the mechanical rigidity to the nucleus^45^. Lamin A couples the nucleus to responses from extracellular stimuli by a complex signaling pathway involving LINC complexes^46^. Compelling evidences have substantiated the fact that A-type lamins are the principal mechanosensitive elements of metazoan nuclei^11^. Previously mechanical properties of the cell nuclei have been studied by several methods including micropipette aspiration, cell compression, particle tracking and AFM^47^,^48^,^49^. Lamin A/C deficient nuclei were proved to be more fluid like^50^. Furthermore, the stiffness of the Xenopus oocyte nuclei significantly increased with the expression of lamin A in a concentration-dependent manner^12^. In retrospect, elasticity and viscosity of the cytoplasm were dramatically reduced in lamin A/C deficient MEF cell lines as revealed by ballistic intracellular nano-rheological experiments^51^. It is a well-documented fact that mechanical properties of several cytoskeleton-based processes like cell motility, coupled MTOC, nuclear dynamics and cell polarization also highly depend on the integrity of the nuclear lamina^51^,^52^. On the other hand, distribution and concentration of lamin A are highly tissue specific^10^. A-type lamins are present in fibroblasts and muscle cells but absent in several blood cells including lymphocytes in inflammatory infiltrates, white pulp in spleen and also in neuroendocrine cells^53^. In other words, soft tissues contain less lamin A compared to the harder counterpart. The thickness of the lamin layer fluctuates with different cell types and their pathophysiological state^54^. This explains why particular tissue types are affected in laminopathies with concomitant variation in nuclear rigidity. Laminopathies can be broadly subdivided into three major categories based on the affected tissues viz. muscular dystrophies, lipodystrophies and neuropathies reflecting a high percentage of lamin A in these tissues^22^. We reasoned to elucidate the mechanism of the pathophysiology of muscular dystrophies by structural hypothesis. Earlier, we investigated how the mutations in lamin A leading to Dilated Cardiomyopathy alter the bulk viscoelastic behavior of the protein which in turn affects the elasticity of the endomyocardium^41^. In our current work, we have shown the origin of elasticity of the lamin A at single molecular level. We investigated the mechanical property of lamin A at the dimer level of fragments of the rod domain. In retrospect, we had also shown for the first time, the elastic properties of Ig-fold globular domain at a single-molecular level in the context of a mutant causing EDMD^55^. Lamin A Ig domain does not have the capacity to form the network alone whereas different Lamin A fragments containing part of the coiled-coil forming rod domain have the capacity to from the short lamin-like structures *in vitro* termed as mini-lamin or half-lamin^26^. It has been shown from electron micrographs that the dimers of different lamin A fragments formed structures similar to those of full-length lamin A^56^. Hence, these fragments were termed as half-lamins or mini-lamins^26^. We also obtained networks similar to that of *in vitro* assembled full-length lamin A as previously observed by Aebi *et al*^56^. However, it must be noted that *in vivo* lamin A assembly cannot be recapitulated entirely by its mode of assembly *in vitro*. The lamina in a cell is a complex network comprising of interactions of lamins with non-membrane bound and transmembrane nuclear proteins^45^. Therefore, the filament structure and its mode of assembly are likely to be modified by proteins like Lap2α, lamin B-binding protein and Lap2β in the cell^57^. Particularly these portions of the rod domain also harbor a vast number of mutations leading to laminopathies. Detailed analysis of the mutations from patient database afflicted with laminopathies reveals that the rod domain is the second most important domain to study after the tail domain in terms of concentration of mutational hotspots (shown in Fig. 6A). Almost 40% of the total laminopathic mutations cluster in the rod domain of which nearly 76% are concentrated in 1B & 2B (shown in Fig. 6). Rod domain of lamin A protein plays an important role in lamin higher order assembly and the network formation. Hence, we considered the lamin 1B and 2B domains for our study. All the intermediate filament proteins form the coiled-coil dimer by interlocking the two alpha helices through the apolar residues at position a and d. But for the ideal heptad b, c and f positions should also be occupied by the charged residues whereas e and g positions should be filled by the lysine and glutamic acids which strongly favors the interchain interaction^58^. For 1B domain, we observed the onset of the heptad repeats from the residue Lys 85 which stretched up to residue Ile 210. This was in good agreement with the lamin A database (http://www.dmd.nl/lmna_home.html) where 1B domain covers 81-218 residues. We noted a seven residue heptad repeat skip from residue 120–126 in the 1B domain from theoretical structural predictions of COILS/PCOILS. Similarly for the 2B domain also, we observed the same four residues (SRRL) insertion at 334–337 positions. This is in agreement with the crystal structure data where four residues insertion was shown at position 327 and was termed as stutter defect^27^.

When we studied the network forming ability of these short lamin fragments to ultra-structural details by scanning electron microscopy, we observed that lamin 1B domain formed a network much similar to full-length lamin A^56^ but 2B domain yielded the short spindle-like structure at similar conditions. This was a novel observation and hitherto not reported. From the scanning electron micrograph, it was clear that 1B domain has the increased propensity to form crisscrossed network compared to 2B domain. Therefore, rod 1B could be designated as the principle component of lamin A as far as the network forming ability is concerned. Hence, any mutation in this domain is most likely to perturb normal network assembly, which might lead to a fragile nucleus.

To explore the mechanical resilience of the 1B and 2B domain we used the SMFS technique. Formation of the dimer at the molecular level plays a pivotal and deterministic role in the higher order assembly of lamins which proceed through the hydrophobic association of the helical rod region. We investigated the mechanical properties of the 1B and 2B domains at their dimer level. Surprisingly 1B dimer was unzipped at slightly lower force (~70 pN) compared to the lamin 2B dimer (~80 pN). Despite the longer length of 1B dimer, the interhelical interactions were comparably stronger for the 2B domain. However, both the domains were unfolding via several intermediate pathways which suggest that they are flexible in nature. End-to-end contour length increase for the 1B domain was ~82 nm which essentially fitted into PCOILS/COILS heptad repeat calculations. Based on these predictions, lamin 1B form coiled-coil dimer from residue 85–210 amino acid and this part contributes to a major extent in the resistance for unzipping of the 1B domain. However, both 1B and 2B domain were unfolded via several pathways. Also, the contour length analysis for both the coiled-coil unzipping events confirmed the presence of stutter in 2B and a heptad skip in 1B. From the heptad repeat analysis, we showed that both 1B and 2B domains possess a heptad skip at 120 position for the 1B and at 334 for the 2B domain. Due to this heptad skip the dimers are mechanically unstable at that position, so in the presence of the unzipping force molecular resistance for the 1B dimer emerged from the residues 85–119 and 127–210 and for 2B it was from residues 305–333 and 338–390. Hence, both the 1B and 2B dimers unfolded via an intermediate pathway. Crystal structure data for 2B also confirmed the heptad repeat skip^27^. The importance of this heptad skip is still unknown. The intermediates could also arise from the inter-helical interactions and then intra-helical uncoiling. Interestingly, 2B was slightly stiffer than 1B despite the variation in their dimer length. Therefore, it can be aptly concluded that the elasticity at the single molecular level was comparable for 1B and 2B domains. In SMFS experiments, we applied a directional force whereas in thermal denaturation thermal energy was uniform in all directions. However, the orientation of the dimer in cells cannot be assigned individually in an array of the crisscross network making up the fibrous lamina. Moreover, stress applied on the nucleus under physiological conditions need not be directional. Hence, this mode of unzipping of the dimers could not be correlated directly to the mechanical instability of the lamina inside the cell. Therefore, the SMFS results would hold true for a scenario where the applied force vector orients across the length of the dimer. Lamin A 1B and 2B dimers are thermally stable up to 72 °C. The thermal unfolding is a bulk property of the molecule. Both the 1B and 2B domains could form higher order structures via self-assembly. So in the aggregated state, 1B domain required more energy to unfold. 1B domain followed three-state unfolding whereas 2B domain unfolded via two-state pathway. However, external mechanical forces are transmitted to the nuclear lamin network when the nucleus is deformed but unzipping of the lamin A dimer is largely governed by the magnitude and the direction of the force, interaction of the other proteins with the lamin A network. Although the unzipping of the lamin dimer would cause reassembly of the lamin network, that causality is not necessarily reversible; the lamina could be reorganized without breaking the dimers. We showed that the elastic nature of the dimer was not changed significantly upon mutation E161K. Similar result was obtained from molecular dynamics simulation studies, where the elastic behavior of the 2B domain was unchanged at the dimer level due to the mutation E358K^59^. Nevertheless, the higher order network assembly and hence the nuclear morphology was significantly perturbed as a result of the mutations K97E & E161K^40^.

In conclusion, we can say that the unfolding behavior of the 1B & 2B domains at the single molecule level as well as bulk level would reflect directly on the viscoelasticity which in turn would be the reason to perturb the rigidity of the lamina, a major hallmark in all laminopathies. To address the viscoelastic behavior, we performed the rheological experiments. Earlier it was shown that full-length lamin A network could resist deformation up to 500% of the strain^41^. We observed that lamin 1B domain itself could resist up to ~200% strain whereas 2B network yielded at 100% strain only. This is in agreement with steered molecular dynamics simulation of the 2B dimer which showed that it could resist deformation up to >150% of tensile strain at 1 m/s pulling speed^59^. Therefore, the elasticity of lamin A protein molecule stems most prominently from the 1B domain which plays a critical role in the polymerization of the molecule also. This importance of the1B domain is supported by the observation that 42% of the total rod domain mutations occur there compared to 24% in rod 2B (see Fig. 6A). Possibly, this is due to the fact that majority of the mutations concentrated principally on 1B produce debilitating effects in patients.

To the best of our knowledge, this work presents the first report in the lamin field characterizing the mechanical behavior of different lamin fragments from the single molecule level to the highly polymerized state. We conclude that the mutations in lamin A, particularly in 1B and 2B, perturb polymerization to a different extent and this might possibly lead to attenuated elasticity of nuclear lamina, which leads deformed and fragile nuclei as observed in the majority of laminopathies.

## Methods

### In silico structure prediction

We investigated the heptad repeats of the lamin 1B and 2B domains using PCOILS/COILS server. We took 71–242 amino acids as 1B which included both the N-terminal and C-terminal linker region and 305–390 amino acids as 2B domain. We used MTIDK matrix and window size as 14.

### Molecular cloning, expression and purification

To apply the mechanical unzipping force to different coiled-coil lamin domains, we used (I27)_3_1B 1 C for 1B and (I27)_3_ 2B 1C for 2B domain. We fused lamin 1B and 2B domain to the three I27 cassettes containing pQE80L vector. We introduced three I27 domains by iterative cloning method as described previously. Forward primer 5′ CGCGGATCCGCGAGCCGCGAGGTGTCCGGCATC 3′ and reverse primer 5′ CGGGGTACCCCGTTAGCAGGAAGATCTTCCCGCCAGCCGGCTCTCAAACTCAC 3′ were used to amplify lamin 1B domain using Phusion High-Fidelity DNA Polymerase (Thermo Scientific Inc., USA). Similarly, for lamin A 2B, 5′ CGCGGATCCGCGCAGCTCAGCCAGCTCCAGAAGC 3′ and 5′ CGGGGTACCCCGTTAGCAGGAAGATCTTCCTAGCCTCTCCTCCTCGCCCTCC 3′ were used as forward and reverse primer respectively. In both the forward primers, BamH1 was used as a restriction enzyme and BglII and Kpn1 sites were introduced in the reverse primers. PCR products of 1B and 2B domains were digested with BamHI-HF and KpnI-HF (New England Biolabs, USA) and gel purifications were performed using Gel Extraction Kit (Qiagen, Hilden, Germany). pQE-80L vector containing three I27 cassettes was similarly digested with BglII and Kpn1-HF restriction enzymes (New England Biolabs, USA) and ligations were done using T4 DNA Ligase (New England Biolabs, USA) at 16 °C overnight. For only 1B and 2B domains, we followed the same path but instead of using pQE80L (I27)_3_ vector we used only pQE80L vector. K97E and E161K were generated as reported earlier^40^. The ligated products were transformed into the XL1-blue cells and positive colonies were selected using colony PCR method. Furthermore, we confirmed the positive clone using Sanger sequencing data. Then all the plasmids were transformed into BL21 (DE3) pLysS cells and induced with 1 mM IPTG at 0.5 OD_600_ for 4 hr at 37 °C. Cell pellets were lysed with 25 mM Tris-HCl (pH 8.5), 250 mM NaCl, 1% Triton-X 100. All the proteins were purified using His-trap column (GE Healthcare Biosciences, USA) followed by gel filtration with Superdex 200 column (GE Healthcare Biosciences, USA) and resolved on 10% and/or 20% SDS-PAGE followed by Coomassie staining. Final proteins were dialyzed using 25 mM Tris-HCl (pH 8.5), 250 mM NaCl buffer. Protein concentrations were checked with Bradford reagent (Bio-Rad, USA).

### Scanning Electron Microscopy (SEM)

All the protein samples assembled in 10 mM Tris-HCl (pH 8.5), 250 mM NaCl, and 1 mM DTT containing buffer were placed on coverslips, dried in vacuum and coated with gold. All the images were taken in Hitachi S530 Scanning Electron Microscope (Japan) between 4000-6000x magnifications at 25 kV.

### Mechanical unzipping of coiled-coil domain

We performed mechanical unzipping experiments using single-molecule force spectroscopy (SMFS) on a custom-built atomic force microscope (AFM) as described previously^60^. All the SMFS experiments were performed at a pulling speed of 1000 nm/s and spring constants of the cantilevers were calculated to be ~35 pN/nm in the buffer. Before each experiment, protein samples were routinely centrifuged at 13000 rpm for 5 min and 2–5 μM proteins were used. All the experiments were carried out in 25 mM Tris-Cl (pH 8.5), 250 mM NaCl buffer at 25 °C.

### Data analysis

All the force-extension (F-X) traces were fitted with the worm-like chain model using the Eq. 5.




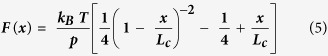




where, p, L, k_B_ and T denote persistence length, contour length, Boltzmann constant and absolute temperature respectively. All the F-X traces were analyzed using Igor Pro 6.02 (Wave Metrics, USA).

### Thermal unfolding studies

Thermal unfolding studies of all the proteins were performed using differential scanning calorimetry (DSC). For these denaturation studies, 100 μM and 20 μM samples were used for lamin 2B and 1B domains respectively. All the experiments were performed in 25 mM Tris-Cl (pH 8.5), NaCl buffer with a scanning rate of 30 °C/hr at ~30 psi pressure in a VP-DSC Microcalorimeter (Microcal, LLC. Northampton, MA, USA). For both the samples temperature ranges were 10–85 °C. All the thermograms were analyzed with in-built VP Viewer software with Origin 7.0. The lamin 2B domain unfolding were fitted with the independent two-state unfolding model whereas lamin 1B domain unfolding was fitted with the independent non-two-state transition models. In the independent two-state model, molar heat capacity varies with the temperature by the Eqs 6 and 8.




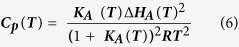




The molar heat capacity in non-two-state transition varies by the Eqs 7, 8, 9.




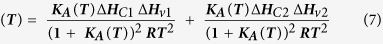







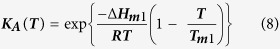







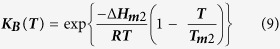




C_p_ (T) is the molar heat capacity; K_A_ (T) and K_B_ (T) are the equilibrium constants for the unfolding events, ∆H_A_ (T) change in the calorimetric enthalpy, T_m_ is the temperature of the transition point, ∆H_m_ molar enthalpy change at the transition point. ∆H_c_ is the change in the calorimetric enthalpy and ∆H_v_ is the van’t Hoff enthalpy. For the two-state unfolding events calorimetric enthalpy is equal to the van’t Hoff enthalpy. The thermograms were fitted using Marquardt methods based on the non-linear least squares guessing the each parameter and then fittings were improved by the several iterations.

### Rheological measurements

Rheological measurements were performed in a rheometer (Rheoplus/32 v3.61, AntonPaar, Graz, Austria) where the lower plate was fixed and shear deformations were applied by the upper cone plate. We applied the shear force on the lamin protein for 1000 sec at a constant 1% strain and at an angular frequency of 5 rad/sec for the build-up experiments. Appropriately diluted the lamin protein samples were applied between the two plates in assembly buffer containing 25 mM Tris-Cl (pH 8.5), 250 mM NaCl at 25 °C. For the build-up experiments, we used 1, 5, 10 and 20 μM of both lamin 1B and 2B protein and 20 μΜ of both the proteins were used for the network yielding experiments where 0 to 500% strains were applied at a constant angular frequency of 5 rad/sec. All these experiments were performed as described previously^41^.

## Additional Information

**How to cite this article**: Bera, M. *et al*. Significance of 1B and 2B domains in modulating elastic properties of lamin A. *Sci. Rep.* **6**, 27879; doi: 10.1038/srep27879 (2016).

## Supplementary Material

Supplementary Information

## Figures and Tables

**Table 1:** Results from the single molecule force spectroscopy of both lamin A 1B and 2B domain.

	Contour Length, (ΔL_c_) (nm)	Unfolding Force, F (pN)	Persistence length, p (nm)
1B (main peak, # events 44)	**82 ± 16**	**67 ± 36**	**0.50 ± 0.25**
1B (intermediate peak, # events 23)	**40 ± 13**	**79 ± 28**	**0.55 ± 0.19**
2B (main peak, # events 49)	**51 ± 15**	**77 ± 28**	**0.39 ± 0.24**
2B (intermediate peak, # events 17)	**25 ± 9**	**76 ± 26**	**0.4 ± 0.2**

**Table 2:** Thermodynamic parameters calculated from differential scanning calorimetry (DSC).

	∆H_cal_ (kcal/mol)		T_m_(°C)	
	∆H_cal1_	∆H_call_	T_m1_	T_m2_
**1B**	55.6 ± 0.5	15.3 ± 0.4	63.4 ± 0.03	72.0 ± 0.1
**2B**	3.7 ± 0.13		61.0 ± 0.1	
