# Supplementary material for: Significance of 1B and 2B domains in modulating elastic properties of lamin A
Source: Sci Rep. 2016 Jun 15;6:27879. doi: 10.1038/srep27879 (PMC4908593; doi:10.1038/srep27879)
Supplement: Supplementary Information [file srep27879-s1.pdf]

Supplementary information

Significance of 1B and 2B domains in modulating elastic properties of lamin A

Manindra Bera[†], Sri Rama Koti Ainavarapu[¶] & Kaushik Sengupta[†*]

[†]Biophysics & Structural Genomics Division, Saha Institute of Nuclear Physics, 1/AF, Bidhannagar, Kolkata-700064, India

[¶] Department of Chemical Sciences, Tata Institute of Fundamental Research, Mumbai, Homi Bhabha Road, Mumbai, 400005, India

***To whom correspondence may be addressed.**

E-mail: kaushik.sengupta@saha.ac.in

Supplementary Fig 1.

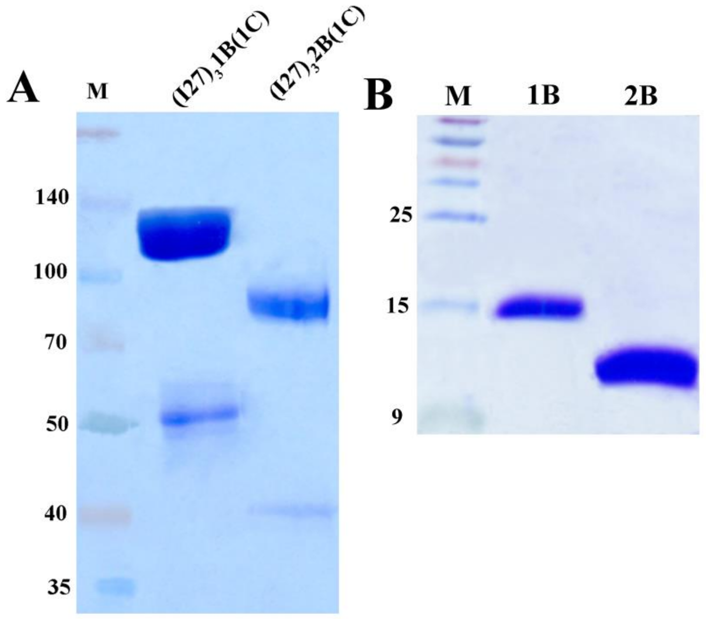

Characterization of the protein using SDS-PAGE and dimer formation of the chimeric protein. A cysteine on the C-terminal end of both the chimeric constructs, $(I27)_3$ 1B (1C) (MW ~ 58 kD) and $(I27)_3$ 2B (1C) (MW ~ 40 kD), was introduced to ensure stable the dimer formation. Panel A represents $(I27)_3$ 1B (1C) and $(I27)_3$ 2B (1C) resolved on a non-reducing 10% SDS-PAGE without DTT and/or ß-mercaptoethanol. Major percentage of both the protein bands appeared in the dimeric position which confirmed the disulphide bond formation. Panel B represents the 1B and 2B monomer (without any I27 construct) in 20% reducing SDS-PAGE.

Supplementary Fig 2.

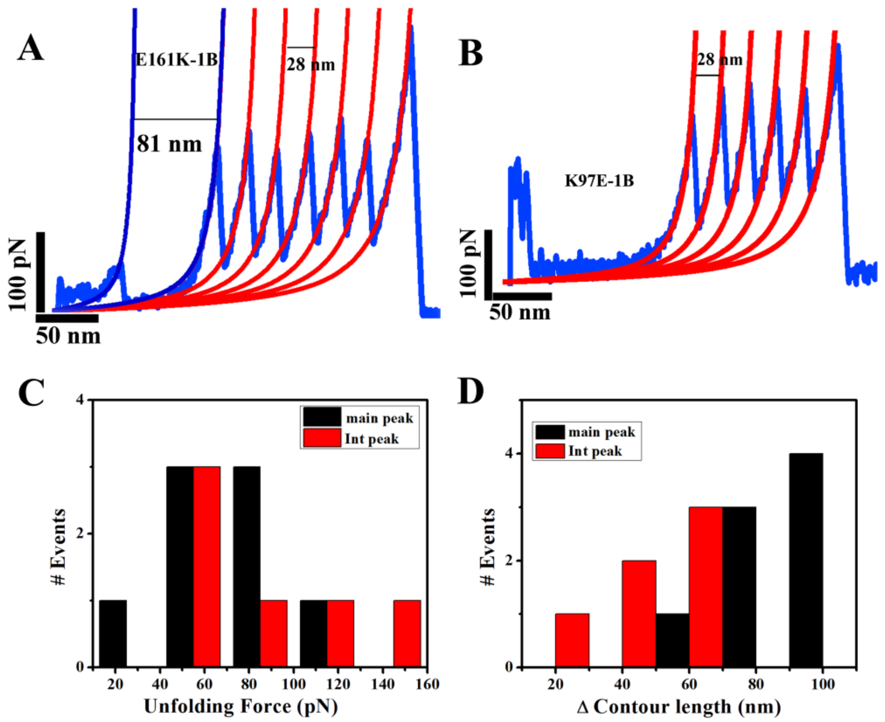

Characteristic unzipping of mutant 1B domains by the single molecule force spectroscopy. Force–extension (F-X) curves for the unzipping of the E161K and K97E dimers were shown in panel A and B respectively. Histograms for the unfolding force and increase in contour length for the mutant E161K dimers were shown in C and D respectively. The unfolding force of the E161K dimer was comparable to the wild type 1B domain (~ 70 pN) and the increase in contour length was ~85 nm. The mutant K97E dimer unraveled at low forces below the noise level. WLC fits were also shown in the F-X curves.

Supplementary Fig 3.

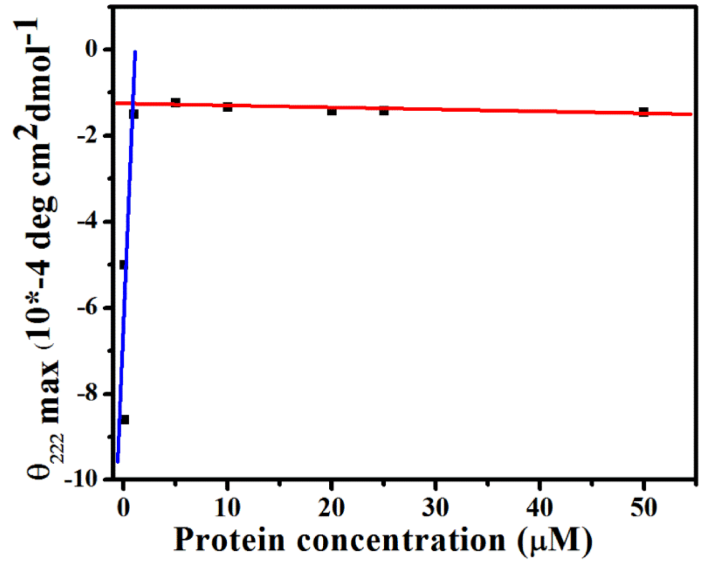

Secondary structural transition of the 2B domain by circular dichroism (CD) study. The inflection or structural transition point of the lamin A 2B domain was calculated using far-UV CD. All the CD experiments were done in Tris-cl (pH 8.5), 250 mM NaCl buffer and the data were recorded from 190 to 260 nm and path length of the cuvette was 0.1 cm. 2B domain showed the predominantly alpha helical structure and the molar ellipticity at 222 nm (Y-axis) was plotted with concentration in X-axis. The inflection or transition point for the 2B was at 0.8 μM concentration which might be due to higher order assembly formation.
